# Muscle defects due to perturbed somite segmentation contribute to late adult scoliosis

**DOI:** 10.18632/aging.103856

**Published:** 2020-09-25

**Authors:** Laura Lleras-Forero, Elis Newham, Stefan Teufel, Koichi Kawakami, Christine Hartmann, Chrissy L. Hammond, Robert D. Knight, Stefan Schulte-Merker

**Affiliations:** 1 Institute for Cardiovascular Organogenesis and Regeneration, Faculty of Medicine, WWU, Münster, Germany; 2 Hubrecht Institute-KNAW and University Medical Center Utrecht, CT, Utrecht, The Netherlands; 3 The School of Physiology, Pharmacology and Neuroscience, Biomedical Sciences, University of Bristol, Bristol, UK; 4 Institut für Muskuloskelettale Medizin (IMM), Abteilung Knochen- und Skelettforschung, Universitätsklinikum Münster, Germany; 5 Laboratory of Molecular and Developmental Biology, National Institute of Genetics, Mishima, Shizuoka, Japan; 6 Centre for Craniofacial and Regenerative Biology, King´s College London, London, UK

**Keywords:** zebrafish, muscle, vertebral defects, adult degenerative scoliosis, aging

## Abstract

Scoliosis is an abnormal bending of the body axis. Truncated vertebrae or a debilitated ability to control the musculature in the back can cause this condition, but in most cases the causative reason for scoliosis is unknown (idiopathic). Using mutants for somite clock genes with mild defects in the vertebral column, we here show that early defects in somitogenesis are not overcome during development and have long lasting and profound consequences for muscle fiber organization, structure and whole muscle volume. These mutants present only mild alterations in the vertebral column, and muscle shortcomings are uncoupled from skeletal defects. None of the mutants presents an overt musculoskeletal phenotype at larval or early adult stages, presumably due to compensatory growth mechanisms. Scoliosis becomes only apparent during aging. We conclude that adult degenerative scoliosis is due to disturbed crosstalk between vertebrae and muscles during early development, resulting in subsequent adult muscle weakness and bending of the body axis.

## INTRODUCTION

As the human population gets older, the effects of aging of the musculoskeletal system and its consequences on the quality of life have become increasingly important to understand. Diseases that result in bending of the spine are more common in elderly populations. Camptocormia (a 45 degree anterior bent of the lower joints of the spine) has an average age of onset of 66 years [1] and from the age of 50 years the prevalence of thoracic scoliosis is 24.2% [2]. Adult scoliosis is a back deformity in a skeletally mature individual [3] with a Cobb angle ≥ 10°, and can be divided into two classes: 1) idiopathic, where the patient has a history of adolescent idiopathic scoliosis, which progresses and worsens with age; 2) *de novo*, without previous symptoms or presence of scoliosis before onset of adult symptoms [4]. *De novo* scoliosis is becoming one of the most common clinical presentations found in the aging spine [5]. A unique genetic explanation for scoliosis has not been identified. However, mutations in genes associated with the Notch-Delta pathway and the segmentation clock genes have been found in some congenital scoliosis patients [6].

**Figure 1:**
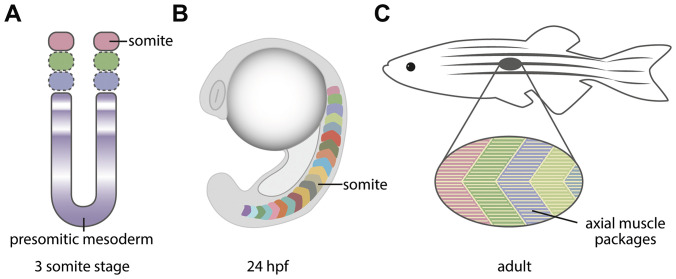
**Graphical depiction of how the segmentation of the presomitic mesoderm leads to the formation of the axial myotome.** (**A**) The oscillation of Notch-Delta genes leads to the segmentation of the presomitic mesoderm into somites. The 3 somite stage is shown. (**B**) The somites are transient structures with a chevron shape in the paraxial mesoderm of the zebrafish embryo, and will generate the axial muscles and later aspects of the vertebrae. (**C**) The adult zebrafish musculature presents the same segmental periodicity as the embryonic somites.

The segmentation clock controls an essential process in the vertebrate embryo and leads to the formation of the somites, transient structures which will later contribute to the formation of one of the most well-known and characteristic structure of all vertebrates, the vertebral column. This structure together with the axial muscles act to control axial posture and function. During early stages of development, the pre-somitic mesoderm is segmented from anterior to posterior by the periodic expression of genes from the Notch-Delta family (Figure 1A). This expression occurs as an oscillatory wave and conveys a clock-like pattern for future segmental boundaries in the presomitic mesoderm. The periodicity of the clock determines when the somites bud off from the presomitic mesoderm and is different for each species [7]. The somites later become compartmentalised into the sclerotome, dermomyotome and syndetome (Figure 1B) which respectively form i) the skeleton, ii) muscle and dermis and iii) tendons and ligaments of the body and limbs. Early myoblasts in the somite arise adjacent to the notochord (adaxial) and express En2 and *myoD* [8, 9]. In chick and zebrafish the adaxial cells expressing En2 migrate laterally and express slow myosin isoforms subsequent to their differentiation in the peripheral region of the future myotome as slow myofibres [10, 11]. More medially located myoblasts will express fast myosin and form fast muscle [12, 13]. Myofibres arising from the dermomyotome expand to the length of each somite and become anchored to the myoseptum, forming the characteristic layered muscle found in zebrafish [14] (Figure 1C).

The importance of axial muscle function in the development of scoliosis is not understood. Human clinical studies have focused on detailed radiographic analyses of the vertebrae in patients with scoliosis [3], but the muscles of these patients have not been well characterised. In humans the spinal musculature can be classified into two major groups: the superficial or extrinsic musculature, which controls limbs and respiratory movements, and the deep or intrinsic musculature (nearest to the vertebral column) which maintains posture and allows movement of the vertebral column [15]. The spinal muscles, in addition to motion functions, are also essential for stabilizing the spine [16]. In patients with idiopathic scoliosis, abnormalities in the multifidus, one of the intrinsic spinal extensor muscles, have been linked to development of the spinal curvature [15, 17]. Asymmetrical differences in trunk strength of adolescent females with scoliosis, has been attributed to weakness in the paraspinal muscles [18]. In addition, some neuromuscular diseases (e.g. cerebral palsy) and paralytic disorders (e.g. polio) can lead to scoliosis due to muscle weakness and imbalance [19]. Muscle imbalances have a stronger influence on bone growth than weight distribution. It has been established that an increase in muscle mass produces stretching of the collagen fibers and periosteum, resulting in local bone growth [20]. Hence, the feedback between muscles and bones reinforces bone defects and muscle imbalance [21]. The interplay between the nervous system and the muscles has also been shown to be involved in the development of scoliosis. *Runx3* mutant mice, which displayed no gross morphological changes in vertebrae or muscles compared to siblings, nonetheless develop scoliosis, due to proprioception loss. Muscle pro-prioceptors are essential in regulating muscle tension and in this way in maintaining a straight axis [22]. Nonetheless, whether disruption to early patterning of the axial musculature can result in scoliosis in adult life has not been investigated.

It has been suggested that human patients with congenital scoliosis may have early abnormalities during somitogenesis [6]. The number of human patients investigated for such associations are low and information on whether disruption in other tissues may contribute to the scoliotic phenotype is lacking. Recently, we described a group of zebrafish mutants for the Notch-Delta pathway and for Tbx6 (*tbx6^-/-^* (fused somite, Fss) single, *her1^-/-^; her7^-/-^ double, and her1^-/-^; her7^-/-^; tbx6*^-/-^ triple mutants). These mutants show varying levels of somite segmentation defects and subsequently mild axial skeletal phenotypes [23]. The structural abnormalities in the vertebrae of these mutants render them a perfect model for congenital scoliosis and enables us to establish the link between early somitogenesis and scoliosis. In the present study, we have analyzed the musculature of the zebrafish mutants *her1^-/-^; her7^-/-^, tbx6^-/-^ and her1^-/-^; her7^-/-^; tbx6^-/-^* at adult and embryonic stages. We found that scoliosis is not coupled to vertebral defects and may correlate with early perturbations of muscle formation during development. Strikingly, *her1^-/-^; her7^-/-^* double mutants develop scoliosis with the same penetrance as wild type animals, despite showing aberrant vertebral skeletal phenotypes. In contrast, loss of Tbx6 function greatly increased the chance of an animal developing scoliosis with age. Finally, we present evidence that this correlates with severe muscle patterning defects during embryogenesis and propose that vertebrae and muscle segmentation are not directly coupled during development.

## RESULTS

### Somite segmentation mutants present vertebral defects with variable levels of scoliosis

**Figure 2:**
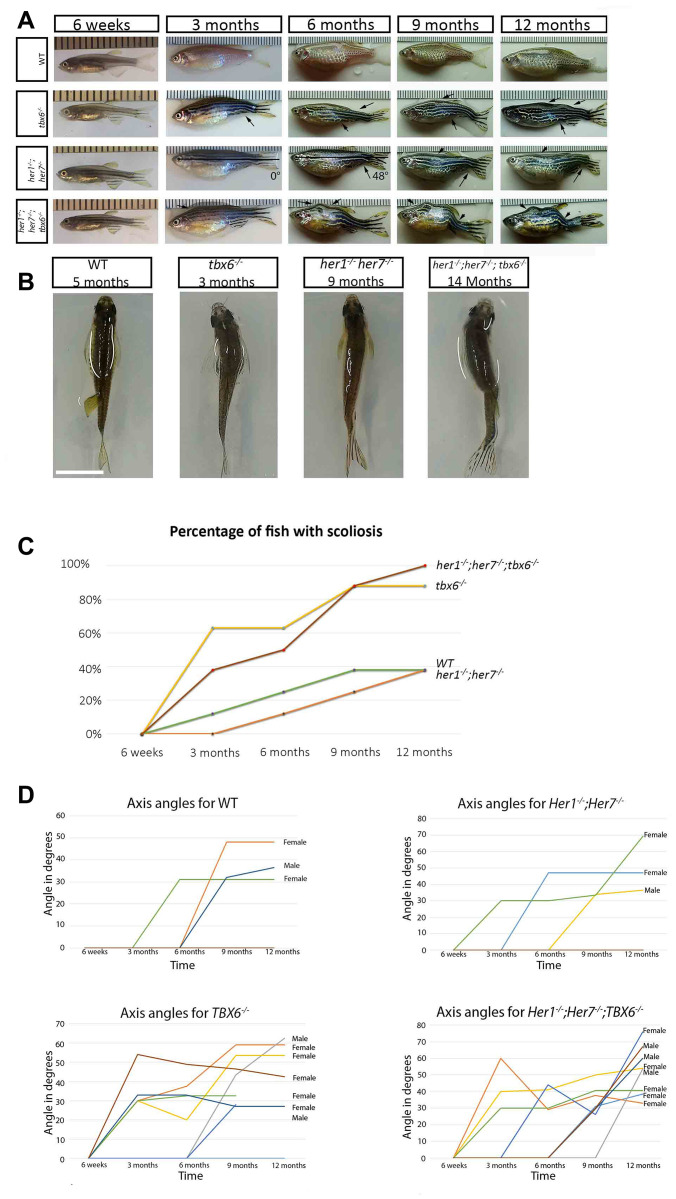
**Clock segmentation mutants develop adult scoliosis.** (**A**) Representative time lapse images of individuals from each genotype over the period from 6 weeks to 12 months, allowing to track the development of scoliosis (arrow). Mutant individuals have already mild signs of scoliosis at 3 months, while wild type exhibit the first indication of deviation from the body axis at 6 months. In the *her1^-/-^; her7^-/-^* individual at 3 and 6 months is an exemplary depiction of how scoliosis measurements were carried out. (**B**) Dorsal view of the different genotypes, showing an S body shape characteristic for scoliosis. (**C**) Graphical representation of the percentage of fish developing scoliosis over time, reaching 100% in the triple mutants and 83% in the *tbx6^-/-^* at the end point. In the wild type and in the *her1^-/-^; her7^-/-^* mutants only 38% presented scoliosis. (**D**) Measurements of axis angles in different individuals at different time points during virtual time-lapse. Only the individuals with an angle of deviation from the body axis are shown in the graph, none bended individuals have an angle of zero. Different line colours represent individual fish. Between 9 and 12 months, two wild type fish (1 with scoliosis and 1 without scoliosis), one *her1^-/-^; her7^-/-^* (with scoliosis) and two *tbx6^-/-^* (both with scoliosis) had to be sacrificed. After the individual was removed, it was still counted as bended or normal in the 12-month quantification. Note: the angle can decrease or increase depending on how the angle of deviation from the body axis develops over time in the individual. The ruler in section A serves as a scale bar, the space between two successive lines marks one millimetre. The scale bar in section B represents 1 cm.

As we have previously shown (Lleras et al., 2018) zebrafish mutants for *her1^-/-^; her7 ^-/-^* and *her1^-/-^; her7^-/-^; tbx6^-/-^* present mild defects in the segmentation of the axial skeleton (fusion and hemivertebrae) with a 100% incidence. This is also true for 80% of *fss* (*tbx6*^-/-^) mutants [23]. Later observation of these three different mutant genotypes revealed that during aging they develop different degrees of scoliosis. In order to record this phenomenon properly, a time-lapse series of photographs were taken of eight mutant and wild type control individuals from each genotype from 6 weeks post fertilization until one year (Figure 2A). Up to the age of 6 weeks there was no phenotypically visible scoliosis. The angle of scoliosis (the angle of deviation from the body axis) was measured from images as described in Materials and Methods (Figure 2C and 2D). In all three zebrafish mutants analysed the first signs of scoliosis could be detected at 3 months post fertilization (Figure 2B–2D).

In order to show that onset of scoliosis occurred at a similar rate in the respective genotypes we evaluated third generation offspring obtained from crosses of those first generation fish used in the initial time lapse. At three months post fertilization 11 out of 35 (31.4%) *tbx6^-/-^* third generation animals developed scoliosis, similar to first generation animals 3 out of 8 (38%) evaluated at a similar age (Figure 2C and 2D). At the same time point, 8 out of 38 (21%) *her1^-/-^; her7^-/-^; tbx6^-/-^* third generation animals presented scoliosis in contrast to first generation animals 5 out of 8 (63%). By the end of the experiment (12 months post fertilization) all *her1^-/-^; her7^-/-^; tbx6^-/-^* first generation animals (8/8) had developed scoliosis. Similarly, in the third generation, all animals (7/7) *her1^-/-^; her7^-/-^; tbx6^-/-^* animals developed scoliosis at 14 months post fertilization (Figure 2D). By 12 months 88% (6/7) first generation *tbx6^-/-^* animals had developed scoliosis (Figure 2C and 2D); this corresponds exactly to the proportion of individuals from these two mutant populations that present vertebrae defects (see Lleras- Forero et al., 2018). Scoliosis in the *her1^-/-^; her7^-/-^; tbx6^-/-^* mutant background was much more severe than in *her1^-/-^; her7^-/-^;* or *tbx6^-/-^* mutants (Figure 2A, 2B and scoliosis angles in Figure 2D). Despite all *her1^-/-^; her7^-/-^* mutants showing vertebral fusions and hemivertebrae, only 5 out of 25 (20%) of the third generation and 38% (3/8) of the time lapse individuals developed visible scoliosis at 12 months post fertilization. All of the *her1^-/-^; her7^-/-^* mutants had vertebral fusions and hemivertebrae in more than one position within the axial skeleton. This value was the same as the wild type scoliosis recurrence. In wild type individuals, scoliosis was first detected at 5 months post fertilization in 1 out of 22 fish (4.5%) and at 6 months post fertilization in 1 out of 8 (12%). At the end of the analyzed period (1 year) 38% (3/8) of wild type animals had developed scoliosis. These individuals had no vertebral defects. Presentation of scoliosis therefore did not correlate with axial skeletal phenotypes in wild type animals. An additional observation, showed that 64% of the females (9/14 that developed scoliosis during the whole experiment) had the first measurable sign of scoliosis at 3 months post fertilization compared to 0 % of males (Figure 2D).

### Muscle volume is altered in somite segment mutants in regions that correspond to spinal curvature

**Figure 3:**
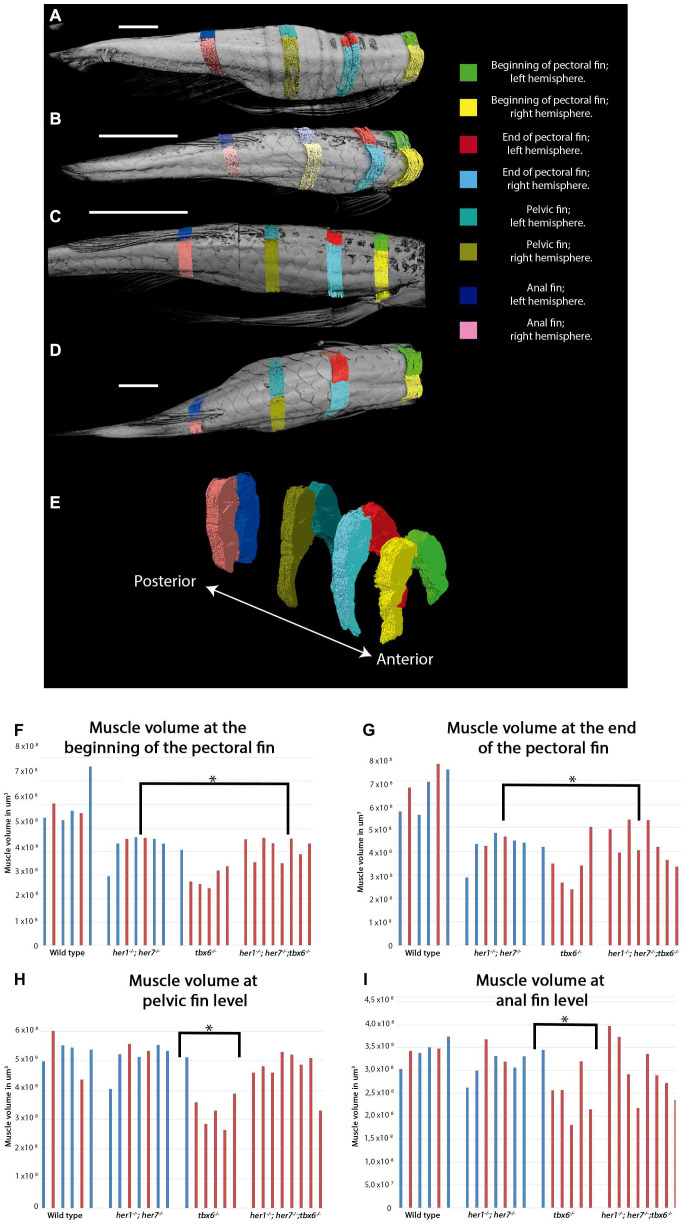
**Muscle volume is affected in mutants at adult stages.** (**A**–**D**) Representative micro CT images showing the dorsal view of fish used for the segmentation of muscles into the left and right side at the four key areas (indicated by the colored regions) in the 4 different groups: (**A**) wild type, (**B**) *tbx6^-/-^* (**C**) *her1^-/-^;her7^-/-^,* and (**D**) *her1^-/-^;her7^-/-^;tbx6^-/-^.* (**E**) Lateral view of reconstructed surface generation of the individual muscle from the WT. (**F**–**I**) graphical results of the volume measurements for every area. (**F**) Muscle volume at the beginning of the pectoral fin. (**G**) Muscle volume at the end of the pectoral fin. (**H**) Muscle volume at pelvic fin level. (**I**) Muscle volume at anal fin level. An asterisk denotes a statistically significant difference between wild type and mutant groups (two tailed significant test P=0,05). Individuals that presented scoliosis during the course of the experiment (represented with red bars) tend to have the same muscle volume as their phenotypically normal siblings (represented with blue bars). The three mutants analyzed have less muscle volume at the first two anterior positions. The muscle of 27 out of 32 individuals could be analyzed to 12 months, because two wild type fish (1 with scoliosis and 1 without scoliosis), one *her1^-/-^; her7^-/-^* individual (with scoliosis) and two *tbx6^-/-^* individuals (both with scoliosis) had to be euthanized between 9 months and one year. After the individual was removed, it was not stained for muscle analysis and therefore will not appear in the graph.

In order to establish if the volume of the muscles in adults carrying mutations affecting somite segmentation was different to wild type animals, all individuals were stained with contrast medium and imaged by micro CT at 12 months of age. The volume of the left and right hypaxial and epaxial muscle of each individual was measured at four positions relative to body landmarks. These landmarks were (1) beginning of the pectoral fin, (2) end of pectoral fin, (3) pelvic fin and (4) anal fin. The first and second positions were selected as the majority of the scolioses were observed in the fish at these levels. The musculature of the fish changes in volume significantly at different anterio-posterior positions, such that anterior myomeres are larger than those in posterior region. By standardizing the locations of all measurements of muscle volume for each animal, we could compare absolute muscle volumes between animals. This allowed us to ensure that any differences in muscle volume were not due to inherent differences in muscle volume along the trunk or due to differences in animal size (Figure 3A–3E). A two-tailed student´s t-test analysis showed that all three mutants have a smaller muscle volume at the beginning and end of the pectoral fin compared to wild type animals (Figure 3F and 3G). In *tbx6^-/-^* mutants, the muscle volume at the pelvic fin and the anal fin was also statistically different from wild type (Figure 3H and 3I). *her1^-/-^; her7*^-/-^ individuals that presented scoliosis during the course of the experiment (Figure 3: red bars in bar chart are scoliotic individuals; each bar is a single individual) tended to have the same muscle volume as their phenotypically normal *her1^-/-^; her7*^-/-^ mutant siblings (Figure 3: blue bars, non- scoliotic individuals; each bar is a single individual). In contrast *tbx6*^-/-^ mutants with scoliosis showed a smaller muscle volume at all anterior positions compared to non-scoliotic *tbx6^/-^* mutant animals. This observation suggests that in *tbx6 ^-/-^* mutants scoliosis correlates with lower muscle volume. No difference was seen between the muscle volume of male and female individuals.

Three-way correlation analysis was performed in order to establish if the length of the fish had an effect on the muscle volume or the angle of deviation from the body axis. It was found that the length of the fish has a negligible correlation with the angle of deviation of the body axis (-0, 26). There is a low positive correlation between muscle volume and fish length (0,47) and a low negative correlation between muscle volume and angle of deviation (-0,39). An interpretation of these correlation scores is that severity of scoliosis correlates with a lower muscle mass but is independent of the length of the fish.

Further analysis of orthogonal sections of the muscle of animals from Micro CT imaging showed that the muscle fibers in the mutants join the vertebrae in a disorganized manner and borders between the myotomes were not distinct in *her1^-/-^; her7^-/-^; tbx6^-/-^* or *tbx6^-/-^* mutants (Supplementary Figure 1). In contrast, in *her1^-/-^; her7^-/-^* mutants, the borders between the individual myotomes were clear but completely disorganized and did not align between the dorsal and ventral sides (Supplementary Figure 1). In addition, animals for all three mutant genotypes possessed cavities in the muscles (arrowheads, Supplementary Figure 1), that were not seen in wild type individuals. Haematoxylin and Eosin staining of transverse sections of wild type and mutant animals clearly revealed disorganization of muscle fibers (Supplementary Figures 1 and 2). In all three mutants analysed, the muscle fibers appeared shorter (data not quantified) and lost their characteristic parallel organization in the myoseptum and the normal transverse morphology at the dorsal and ventral sides. In addition, in all three mutant groups, the shape of the fibers appeared to be variable (Supplementary Figure 2).

### Fast muscle area and fiber size is affected at embryonic stages in *her1^-/-^; her7^-/-^* mutants

Myotome boundaries and the axial skeleton were characterised at 15 days post-fertilization (dpf) (Supplementary Figure 3) to determine whether segmentation defects persist and whether this correlates with axial skeletal defects (axial skeletal defects are indicated by arrows). In all mutants studied, the myotome boundaries were disorganized compared to wild type controls at 15 dpf. Furthermore, in the *tbx6^-/-^* and the *her1^-/-^; her7^-/-^; tbx6^-/-^* mutants, there was a decrease in the number of cells expressing the myotome boundary marker (gSAIzGFFM1954A) (Supplementary Figure 3). Therefore, myotome boundary defects persist throughout development in the whole zebrafish body axis and not only where axial skeletal defects are present.

**Figure 4:**
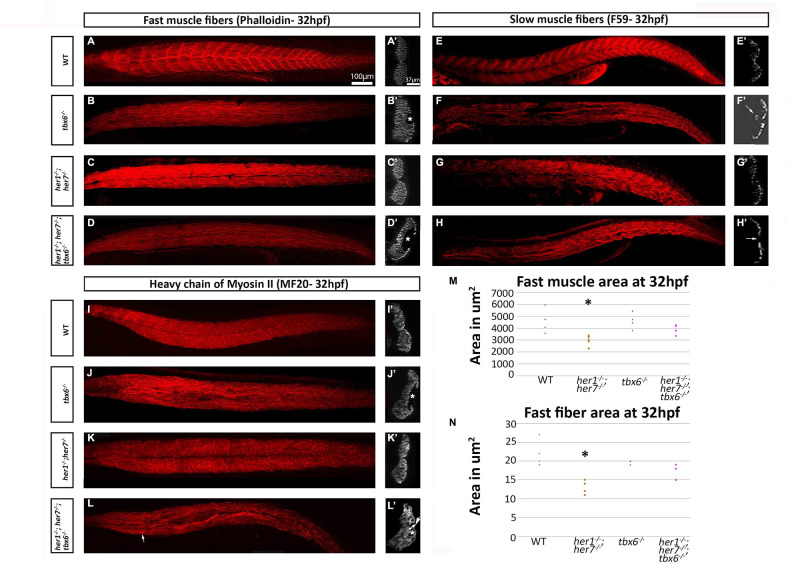
**Fast and slow muscle fibers are affected in mutants at embryonic stages.** (**A**–**D**) Phalloidin staining for fast muscle fibers in all mutants show loss of the characteristic metameric structure. (**A**) In addition, the transverse plane sections at the cloacal level in *tbx6^-/-^* and *her1^-/-^; her7^-/-^; tbx6^-/-^* display hollow cavities where no muscle fibers were present (**B´** and **D´**). F59 staining for slow muscle fibers (**E**–**H**) also demonstrates loss of the characteristic metameric patterning in the mutants. *tbx6^-/-^* and *her1^-/-^; her7^-/-^; tbx6^-/-^* mutants present fusions of the fibers and cavities (**F**, **F´** and **H´**). The *her1^-/-^; her7^-/-^* slow fibers resemble wild type fibers in sections (**G´**). The marker for striated muscle, MF20 (**I**–**L**) shows the same phenotype as phalloidin and allows the visualisation of aggregates of the fibers near the areas where there are lesions in the *her1^-/-^; her7^-/-^; tbx6^-/-^* mutants (arrow in **L´**). Quantifications of fast muscle area (**M**) and fiber cross-sectional area (**N**) at 32hpf display a statistically significant decrease (p= 0.03 and p= 0.009 respectively) of these two criteria in the *her1^-/-^; her7^-/ -^* mutants. In both figures, 6 points were measured. An asterisk denotes when the difference between the wild types and mutants is statistically different (p<0.05).

To investigate further the early muscle phenotypes of these mutants, 32 hours post fertilization (hpf) embryos were analyzed. In all mutants the fast (Phalloidin positive) and slow (F59 positive) muscle fibers lose their metameric organization (Figure 4A–4L). In addition, in the *tbx6^-/-^* and the triple *her1^-/-^; her7^-/-^; tbx6*^-/-^ mutants cavities can be seen in the fast muscle (Figure 4B´, 4D´). These can vary in size and number (Figure 4L´). In *tbx6^-/-^* mutants, cavities were found in 15 sections out of 20 sections analyzed. 30 cavities (between 1 and 5 per section) could be measured with an average size of 1.105μm ± 0.66. In *her1^-/-^; her7^-/-^; tbx6^-/-^* cavities were found in 18 out of 20 optical sections, 40 cavities (between 1 and 5 per section) were measured with an average size of 1.407μm ± 0.93. In order to determine whether the cavities contained cells that do not express muscle fiber markers, whole mount DAPI staining was performed. In all *her1^-/-^; her7^-/-^; tbx6^-/-^* and *tbx6^-/-^* embryos imaged at 32hpf (n=8 for each genotype), the cavities did not contain any cells (Supplementary Figure 4). In addition, the *her1^-/-^; her7^-/-^; tbx6^-/-^* mutants present an agglomeration of MF20-labeled fibers around the vacuolated regions (Figure 4L´). The slow fibers in the *tbx6^-/-^* and the triple *her1^-/-^; her7^-/-^; tbx6^-/-^* mutants appeared to be fused and thicker than in the wild type. In several of these animals there were regions of the slow muscle in which no fibers were present (Figure 4H´) or the slow fibers appeared to invade the inner region containing fast muscle fibers (Figure 4F´) as previously described in *fss* mutants [24].

Quantification of the cross-sectional area of the fast muscle was performed from optical sections. This analysis showed that the *her1^-/-^; her7^-/-^* mutants have a significantly smaller region of fast muscle compared to the wild type at 32 hpf (Figure 4M). Individual fast muscle fiber cross-sectional area was also decreased at 32hpf in *her1^-/-^; her7^-/-^* mutants (Figure 4N). Fast fiber cross-sectional area is furthermore reduced in *tbx6^-/-^* and *her1^-/-^; her7^-/-^; tbx6^-/-^* mutants, but the difference is not significant when compared to wild type animals.

## DISCUSSION

Scoliosis affects an increasing proportion of elderly people, 68% of healthy adults over 60 years of age [25] and 8.85% of adults over the age of 40 years [26]. Despite this high prevalence in aging individuals, the underlying cellular and molecular basis for the majority of cases remains unknown. It is widely thought that scoliosis can arise due to skeletal deformities as a result of aging and that this subsequently affects the muscle. The potential for an embryonic origin for scoliosis has been previously considered [6] and mutations in several genes that are known to be important for segmentation of the body, including muscle and skeleton, have been found in scoliotic patients. These include several genes involved in the somite clock such as the Notch receptor DLL3 [27, 28], the T-box gene TBX6 [29], MESP2 [30], HES7 [31, 32], LFNG [33] and RIPPLY2 [34].

In order to understand more about the link between the somite clock and adult degenerative scoliosis, we analysed zebrafish mutants defective for three clock segmentation genes: *her1^-/-^; her7 ^-/-^,* *tbx6^-/-^* and *her1^-/-^; her7^-/-^; tbx6^-/-^*. These three mutants present mild defects in the axial skeleton (vertebral fusions, hemivertebrae and smaller additional vertebrae), but only start to develop scoliosis as they age (after six weeks of age). In the *her1^-/-^; her7 ^-/-^* mutants there is no correlation between the position or amount of vertebral defects and the risk of developing scoliosis. If vertebral defects were correlated with scoliosis, we would predict that all animals with significant vertebral aberrations would present scoliosis. There is a high incidence of vertebral defects in *her1^-/-^; her7^-/-^* mutants ranging from 1-12 defects per animal at 25 dpf [23] and between 1-4 defects in adults (n=9). If vertebral defects were associated with scoliosis it would therefore be expected that *her1^-/-^;her7^-/^-* mutants should show a high incidence of scoliosis. However, the incidence of scoliosis was the same as in the wild type animals examined at a similar age. When evaluating whether muscle morphology in *her1^-/-^; her7^-/-^* mutants was related to where scoliosis occurred along the body axis we did not observe any difference in muscle volume at regions where scoliosis occurred relative to other axial positions. Furthermore, there were no lesions in fast or slow muscle during embryogenesis in *her1^-/-^; her7^-/-^* mutants. In contrast, *her1^-/-^; her7^-/-^; tbx6^-/-^* mutant animals showed a lower muscle volume in regions in which scoliosis was observed and also had perturbed slow and fast muscle development.

Despite differences in adult muscle phenotype and embryonic muscle development between *her1^-/-^; her7^-/-^* and *her1^-/-^; her7^-/-^; tbx6^-/-^* mutants this was unrelated to the skeletal phenotype. The higher scoliosis incidence in the *her1^-/-^; her7^-/-^; tbx6^-/-^* mutants is probably due to the muscle defects caused by the loss of Tbx6 activity. It is possible, that *her1^-/-^; her7^-/-^* mutants recover from early muscle patterning defects by compensatory growth, but in an absence of Tbx6 function, this cannot occur. In *tbx6^-/-^* and *her1^-/-^; her7^-/-^; tbx6^-/-^* mutants the muscle contains many cavities that originated from embryonic stages. There is no recovery of the early muscle defects in these mutants, potentially due to a deficit in the myogenic precursor population at embryonic stages, in turn due to the role of Tbx6 in regulating differentiation of dermamyotome progenitor cells [24]. In the forming somite Tbx6 function is regulated by Ripply1, a regulator of the somite clock and its expression dictates whether myoblasts undergo differentiation or are maintained in a progenitor state [35]. Thus, perturbation of myogenesis due to loss of Tbx6 function, combined with mutations in *her1* and *her7*, results in long lasting muscle phenotypes that appear to be correlated with scoliosis. The occurrence of muscle phenotypes in compound *her1^-/-^; her7^-/-^; tbx6^-/-^* mutants occurs independently of skeletal abnormalities as there is no correlation of skeletal abnormalities relative to the presence of scoliosis between genotypes.

In humans, spinal muscles are essential for stabilising the spine, and changes in muscle mass have been linked to axis curvatures. It is known that upon spine curvature, muscles adjacent to the curvature are generally weaker and progressively deform [16, 19]. Our results show that this is also true for adult zebrafish in which a decrease in muscle mass was seen adjacent to, and immediately at the scoliotic position, independent of fish age and length. In the clock segmentation mutants presented in this paper the muscles lack segmentation, contain cavities and show a decrease in cross-sectional area early in development. The effect on the muscle is seen before there were any apparent vertebrae segmentation or ossification defects. From early stages of development there is a deficit in the muscles of these mutants. Potentially this results in an impaired ability to stabilise the spine and could contribute to the development of scoliosis. The only *tbx6^-/-^* mutant individual in this study that did not develop scoliosis had higher muscle mass than its scoliotic siblings (similar levels than the wild type and the *her1^-/-^; her7 ^-/-^*) implying that muscle mass and scoliosis are directly correlated. In human patients, differences between the left and right-side muscles at the position of axial curvature have been documented. In the bent side the muscle becomes overstretched, while in the opposite side the muscles are shorter and tighter [21]. We did not observe this in zebrafish, where muscles on both sides of the scoliotic position were equally affected. We note that histological analysis of the zebrafish axis muscles revealed a tendency for the myofibers to be disorganized and smaller in the mutants compared to wild type animals (Supplementary Figure 2). In patients with idiopathic scoliosis, the concave muscle Type I fibers are mildly atrophic and smaller, similar to our findings from zebrafish clock gene mutants [36, 37].

In elderly patients there is no difference in scoliosis prevalence between males and females [26]. In our study, all mutant zebrafish female animals developed scoliosis three to six months earlier than their male counterparts (Figure 2). It has been reported that under laboratory conditions zebrafish reach sexual maturity within 3 months [38]. Sexual maturity in females is associated with a decrease in muscle mass and an increase in fat content. This potentially diminishes muscle strength and thereby contributes to conditions that could lead to scoliosis [39]. The differences observed in the number of scoliotic individuals in the time-lapse compared to the later generation in *her1^-/-^; her7^-/-^; tbx6^-/-^* and *tbx6^-/-^* individuals can be explained by differences in total number of individuals quantified. Regardless, in both generations, there is a trend towards scoliosis, which is much higher than in wild type.

Our data show that loss of *tbx6* function leads to the strongest scoliotic phenotype. The *tbx6 ^-/-^* mutant zebrafish and human patients with mutations in *TBX6* have similar skeletal phenotypes including hemivertebrae, butterfly vertebrae and rib abnormalities [40]. In humans the defective vertebrae are localized at the lower region of the spine [40]. In zebrafish we could not localize the defects to a specific region of the axis. Unfortunately, no information about the muscles of scoliosis patients with mutations in *TBX6* is available for comparison. Our findings from zebrafish intriguingly suggest that scoliosis-associated genes may be needed for normal muscle patterning at early embryonic stages and it is the disruption of early muscle organisation that may underlie scoliosis onset later in life.

The fast muscle domain and fast fiber cross sectional area were decreased in *her1^-/-^; her7 ^-/-^* mutants but not in *tbx6^-/-^* or *her1^-/-^; her7^-/-^; tbx6^-/-^* mutants. Nonetheless, in *tbx6^-/-^* and the triple *her1^-/-^; her7^-/-^; tbx6^-/-^* mutant embryos, the slow and fast muscle fibers show cavities depleted of nucleated cells (Figure 4 and Supplementary Figure 4), which cannot be repaired during development and can still be found in adults (Supplementary Figure 1). Adult *her1^-/-^; her7^-/-^* mutants also present cavities in the muscle, but these were less frequent and smaller. Histological analysis of muscle biopsies of scoliotic patients has shown large amounts of connective and adipose tissue between the muscle fibers, but cavities have not been reported [41]. This phenotype is not common and only in *Drosophila* mutants for the myogenic repressor gene, *holes in the muscles* (*Him*), is a similar phenotype described [42]. Him inhibits myogenic differentiation through the transcription factor Mef2 by direct interaction with Twist [43]. It is known that transcription of *Him* is regulated by Notch signalling [44]. Interestingly, Her1, Her7 and Tbx6 are direct regulators of the Notch-Delta signalling pathway in mammals [45, 46]. This would point to a possible mechanism in which Her1, Her7 and Tbx6, through the Notch-signalling pathway, would regulate myogenic differentiation and lead to the muscle phenotype seen in these mutants. We note that *tbx6^-/-^* mutants have a more than 50% reduction in the number of Pax3+ Pax7+ muscle progenitor cells at embryonic stages and unusually large fast muscle fibres that persists until later larval stages [24]. In addition, in zebrafish it has been clearly shown that *tbx6* interacts with *Mesp-b* and *Ripply1* to regulate myogenesis in zebrafish [35]. Hence, not only segmentation of the muscle is affected in the clock segmentation mutants, but apparently, there is also an effect on muscle differentiation.

In summary, we have shown that the zebrafish mutants for *her1^-/-^; her7^-/-^, tbx6^-/-^* and triple *her1^-/-^; her7^-/-^; tbx6^-/-^* can serve as a model for adult scoliosis, because they reproduce the muscle and bone indicators present in clinical patients. Previously, it has been proposed that human patients with congenital scoliosis, may have early abnormalities during somitogenesis [6] and mutations in components of the Notch- Delta pathway have been shown to cause scoliosis in humans. Our work supports a potential origin for scoliosis as a condition that develops as a consequence of a perturbation to the interplay between the axial skeleton and associated muscles due to perturbations of muscle structure and function. It also highlights how changes to adult muscle volume, and hence strength, occur as a consequence of earlier embryonic perturbations and thus may underlie later, adult musculoskeletal perturbations.

## MATERIALS AND METHODS

### Animal procedures

The work was carried out at the Hubrecht Institute (the NL), according to local laws. Ethical approval was obtained through the relevant DEC committee (HI 10.1801). Standard husbandry conditions applied, according to FELASA guidelines [47]. Embryos were kept in E3 embryo medium (5 mM NaCl, 0.17 mM KCl, 0.33 mM CaCl_2_, 0.33 mM MgSO_4_) at 28°C. For anesthesia, a 0.2 % solution of 3-aminobenzoic acid ethyl ester (Sigma), containing Tris buffer, pH 7, was used.

### Transgenic lines and genotyping

The *fss^sa38869^* and *her7^hu2526^* mutants were provided by Jeroen den Hertog (Hubrecht Institute). The gSAIzGFFM1954A line was obtained by a gene trap method [48]. Double mutants for *her1* in the *her7*^hu2526^ background, were generated as described in Lleras-Forero et al. [23]. DNA was isolated from fin biopsies (AZ 81-02.05.40.19.044) and from embryos. Genotyping was performed as described in Lleras-Forero et al. [23].

### Alizarin Red bone and Hematoxylin and Eosin staining

Hematoxylin/eosin (H&E) was performed on sagittal and transverse cryo-sections (15μm) of adult zebrafish according to standard procedures. H&E sections were photographed on a Nikon eclipse NI with a DS-Ri2 camera and 4x Plan Fluor objective. Alizarin Red staining of bone was performed as described previously [49]. Whole mount stained fish were documented on an *Olympus SZX16* stereoscope with a Leica DFC450C camera.

### Whole mount immunostaining

32 hpf embryos were dechorionated and fixed in 4% PFA overnight. Phalloidin (Invitrogen Alexa Fluor 546) staining was performed according to Goody, 2013 [50]. Staining with MF20 antibody (supernatant) (Developmental Studies Hybridoma Bank, university of Iowa) (1:20) was performed overnight. Embryos were first permeabilized in ice cold acetone for 20 minutes and treated with proteinase K for 30 minutes at room temperature. Secondary antibody (Dianova donkey anti mouse conjugated Cy3) was used at a concentration of 1:250. For the F59 antibody (supernatant) (Developmental Studies Hybridoma Bank, University of Iowa) (1:20), embryos were treated with 3% hydrogen peroxide for 1 hour on ice, followed by permeabilization and proteinase K treatment as described above. Samples were incubated with primary antibodies overnight. Labelling with the F59 antibody utilised amplification with the Tyramide system (PerkinElmer Inc.) by combining detection with a secondary antibody (goat anti mouse IgG/IgM HRP (Millipore)) (1:250) and the TSA ^TM^ plus Cyanine 3 system (PerkinElmer, Inc). For detection of nuclei DAPI was used in which embryos were fix at 32 hpf as described above and then washed twice for 10 minutes in PBS. Staining was performed with DAPI solution (Roth, final concentration of 0.2μg/μl) in Eppendorf tubes, at 4 degrees in the dark, over two nights.

Image acquisition was performed by embedding embryos in 0.8% agarose and visualising on a Leica SP8 confocal microscope using a 20X objective (N.A.= 0,75) for the whole mounts and a 40x objective (water immersion, N.A. =1,0) for the cloacal area acquisitions.

### Photographic record of scoliosis

Embryos from mutant crosses were kept in E3 at 28°C until 7 dpf. They were then housed at a density of 50 embryos in a 3 Liter tank. At six weeks post fertilization, eight juvenile animals, with fully developed swim bladder, from either mutant or wildtype embryos were housed individually in the animal facility. Individuals were fed tetrahymena in combination with Gemma 75 for the first two weeks, followed by Artemia and Gemma 150 for the following two weeks. The same day each individual was anesthetized (as described above) and photographed using an *Olympus SXZ16* stereomicroscope (1.5X PlanApo objective) connected to a DFC450C Leica camera. Immediately afterwards, the individual was returned to warm E3 media without anesthesia. Test subjects were returned to their specific tank in the animal facility only when they were completely awake and moving. This procedure was repeated at 3, 6, 9 and 12 months after fertilization. From 3 months onwards, a picture was taken using a Sony Xperia mobile. Sedation and photography did not take more than two minutes per animal and did not compromise survival.

### Micro-CT imaging and muscle volume measurement

One-year-old fish were fixed in 4% paraformaldehyde overnight at 4 degrees; afterwards an incision was made in the abdomen and the organs were removed. Staining for soft tissue visualization with Micro CT was performed according to Descamps et al., 2014 [51] for 9 days. After staining, the samples were placed in 30% methanol for at least 24 hours. For visualization, the samples were embedded in 1% agarose inside 15 ml Falcon tubes. For Figure 2 the samples were imaged using the Bruker SKYSCAN 1272 micro-CT system. Several scans were conducted for each specimen in a batch, to ensure that the complete specimen was imaged with sufficient optical resolution. Isotropic voxel size was set to 9 mm, with 60 KeV X-ray energy, 50 W current and a 0.25 mm aluminium filter. 1501 projections were collected during a 180^°^ rotation, with 400 ms exposure time. Reconstructions were performed using NRecon (Version 1.7.1.0). Each individual scan overlapped its neighbouring scans in its respective batch by 200 mm, to ensure robust concatenation. For Supplementary Figure 2 the Micro CT scanning was done using the Skyscan 1176 at 65 kV, 385 μA, 1mm Al filter, 0.5° rotation steps, an image pixel size of 8.52 μm and exposure set to 1065 ms. Reconstruction of sections was carried out with NRecon v1.6.10.4.

Reconstructions were processed using ImageJ/Fiji [52]. Concatenation was performed by hand, by identifying the overlapping regions between scans and copy-pasting the batch data into the same folder. Using a custom macro (see below), each concatenated reconstruction was processed into 10-slice (90 mm) thick ‘virtual thin sections’ [53] using the “z projection” tool. This aided interpretation of muscle and increased the throughput of analyzed projections by dividing the number of effective slices per-specimen by ten. Processed stacks of virtual thin sections were then analyzed in Avizo (version 9.0; Visualisation Sciences Group). The specimen was viewed using the “Volume rendering” tool and segmented using the “Edit New Label Field” tool. For every specimen, the epaxial/hypaxial musculature was isolated and segmented in four regions (Figure 2A). Each region was divided into its right and left hemisphere, and the muscle was segmented for 10 virtual thin sections (90 mm). Volumes of every region were then calculated using the “Material statistics” tool. The statistical significance of the data was measured using a two-tailed T-test with unknown variance in Excel 2016.

### Muscle area, fiber size and cavity size quantification in 32 hpf embryos

Using a Leica SP8 confocal with a 40x plan Apo objective (water immersion, N.A. =1,0) and a Z-step size of 0.27μm, an image from the cloacal region from six 32 hpf embryos (stained with phalloidin), was taken. Five slides within each image were randomly chosen and the area was measured manually using Fiji. The data point plotted in the graph is the mean of the 5 slides within one embryo.

Cross-sectional area quantification was done in the images of the cloacal region described above. Using Fiji, 5 randomly selected fibers were manually measured in the slides described above. Each point in the graph represents one embryo (25 different fibers). The statistical significance of the data was measured using a two-tailed Student’s t-test with unknown variance in Excel 2016 (Microsoft).

Cavity size in *tbx6^-/-^* and *her1^-/-^; her7^-/-^; tbx6^-/-^* were measured in the optical sections stained by Phalloidin as described above. Using Fiji, the longest vertical distance of each cavity was measured in μm. The average and standard deviation were calculated for each mutant.

### Scoliosis angle measurements in photographs

In order to measure accurately if individual zebrafish had developed scoliosis and if the scoliosis progressed over time, the individual pictures from the time lapse were used. A straight line was drawn from the tip of the premaxilla, through the middle of the eye to the caudal fin. In fish lacking scoliosis or with a perturbation of the body axis the line ends exactly at the apex of the caudal fin. In a fish with a bent body axis, the line deviates from this position. The angle between the position of the line coming from the premaxilla to the new position of the apex was manually measured. In humans, the spine is considered deformed when the Cobb angle is ≥ 10 degrees [3]. The same criteria were considered for classifying the angle of deviation of the body axis for zebrafish.

## Supplementary Material

Supplementary Figures

Supplementary Custom Macros
