# Supplementary material for: Muscle defects due to perturbed somite segmentation contribute to late adult scoliosis
Source: Aging (Albany NY). 2020 Sep 25;12(18):18603–21. doi: 10.18632/aging.103856 (PMC7585121; doi:10.18632/aging.103856)
Supplement: Supplementary Figures [file aging-12-103856-s002..pdf]

SUPPLEMENTARY FIGURES

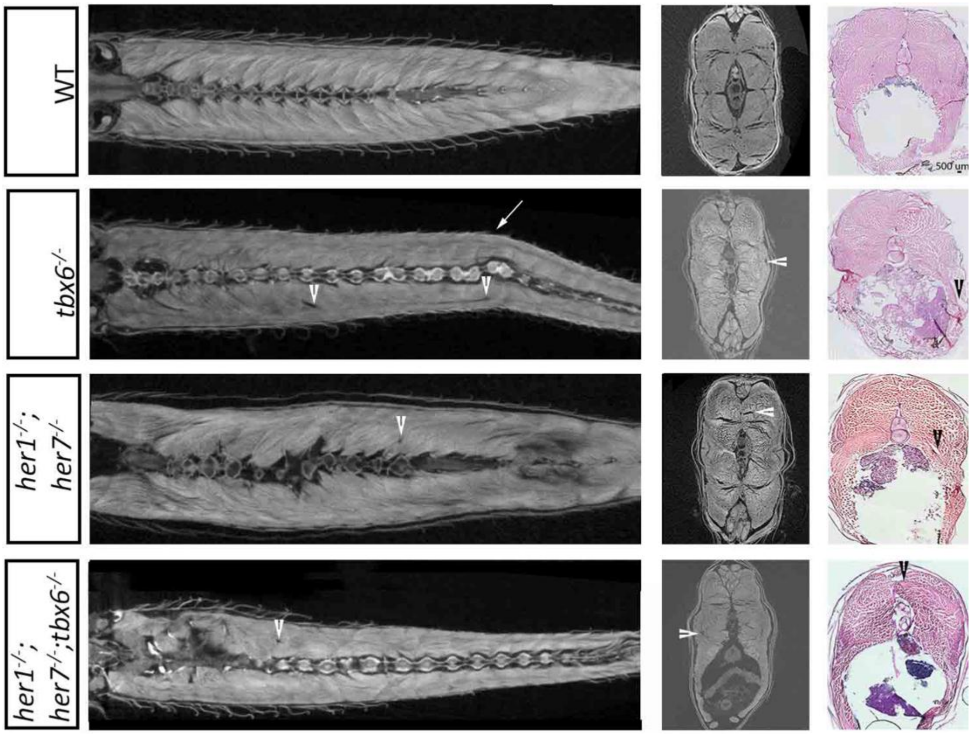

Supplementary Figure 1. Muscles do not join properly to vertebrae and present lesions and a disorganized pattern in adult zebrafish. Micro CT coronal sections of wild type (WT) fish show that the muscles connect to the vertebrae in a stereotypic and organized manner. Individual myotomes can be distinguished. On the contrary, in the different mutants, connections to the vertebrae are disturbed and the individual muscle segments cannot be distinguished. Lesions in the muscle can also be seen in coronal and transverse sections (arrow head). H & E staining of transverse sections of wild type (WT), *tbx6*$^{-/-}$, *her1*$^{-/-}$; *her7*$^{-/-}$ and *her1*$^{-/-}$; *her7*$^{-/-}$; *tbx6*$^{-/-}$ show that muscle fibers are disorganized, less compact and present cavities (arrowhead). The arrow points to a scoliotic region.

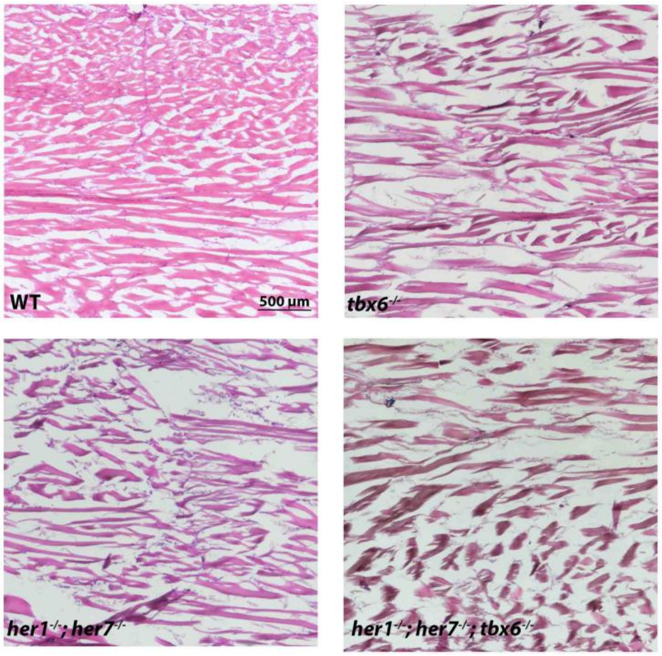

Supplementary Figure 2. Muscle fibers in mutant adults are disorganized, shorter and variable in shape. Sagittal sections of 12-months old zebrafish at the level of a myotome boundary (cloacal level). In all mutant genotypes, muscle fibers are disorganized; fibers vary in size and shape, and their density is decreased when compared to wild type animals.

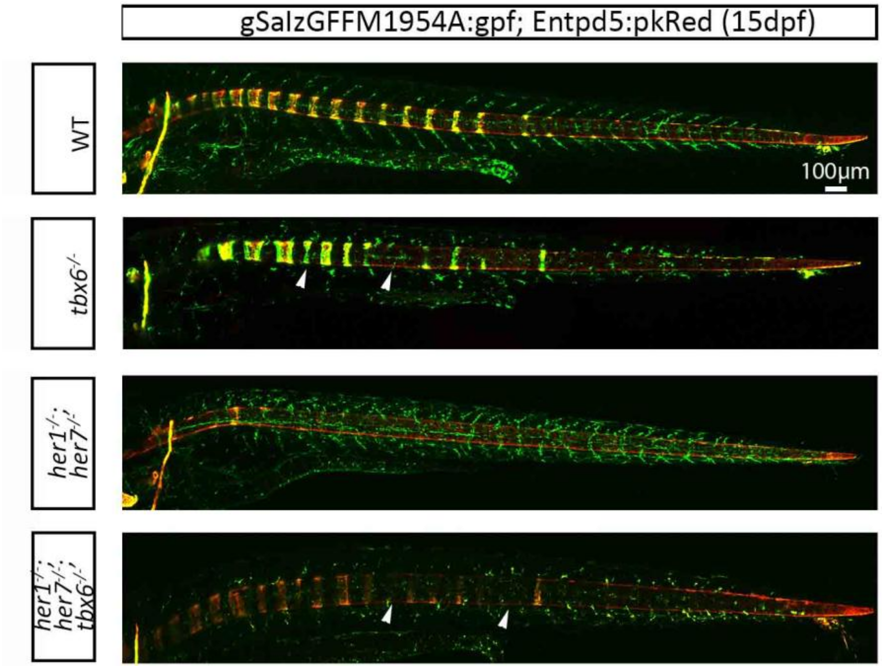

Supplementary Figure 3. Myotome boundaries are affected in mutants. At 15dpf, segmentation defects, which had been detected at the earlier time point are still visible in the myotome boundaries along the whole axis of the mutants as shown by gSAIzGFFM1954A:GFP expression. These occur independently of the axial skeletal segmentation defects (shown by the arrowhead), demostrated by Entpd5:pkRed expression marking the chordacentra.

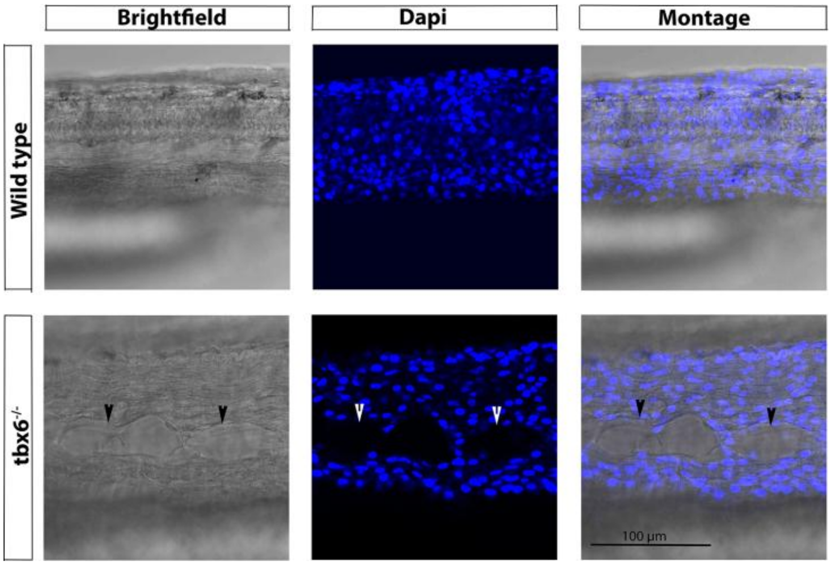

Supplementary Figure 4. The cavities in the muscle of the somite clock mutants are depleted of nucleated cells. Whole mount DAPI staining of embryos at 32hpf shows that the cavities (arrow heads) are devoid of nucleated cells.
