# Supplementary material for: Muscle defects due to perturbed somite segmentation contribute to late adult scoliosis
Source: Aging (Albany NY). 2020 Sep 25;12(18):18603–21. doi: 10.18632/aging.103856 (PMC7585121; doi:10.18632/aging.103856)
Supplement: Supplementary Custom Macros [file aging-12-103856-s001..pdf]

SUPPLEMENTARY CUSTOM MACROS

```
for(i=10; i<=3000; i=i+10){
print(i);
run("Image Sequence...", "open=[“%FIRST IMAGE OF EXAMPLE DATASET.tiff%”] number=10 starting="+i+" increment=1 scale=100 file=[] or=[] sort");
run("Z Project...", "projection=[Sum Slices]");
saveAs("Tiff",”%INTENDED FOLDER FOR VIRTUAL THIN SECTION DATA%”);
close();
close();
}
```
